# Supplementary material for: Structure of the lysosomal mTORC1–TFEB–Rag–Ragulator megacomplex
Source: Nature. 2023 Jan 25;614(7948):572–9. doi: 10.1038/s41586-022-05652-7 (PMC9931586; doi:10.1038/s41586-022-05652-7)

nature portfolio

https://doi.org/10.1038/s41586-022-05652-7

Supplementary information

Structure of the lysosomal mTORC1–TFEB–Rag–Ragulator megacomplex

In the format provided by the authors and unedited

Supplementary Fig. 1: Original images of SDS-PAGE and immunoblots.
Cropped regions shown in the main figures and extended data figures are indicated with dashed lines.

Fig. 1a

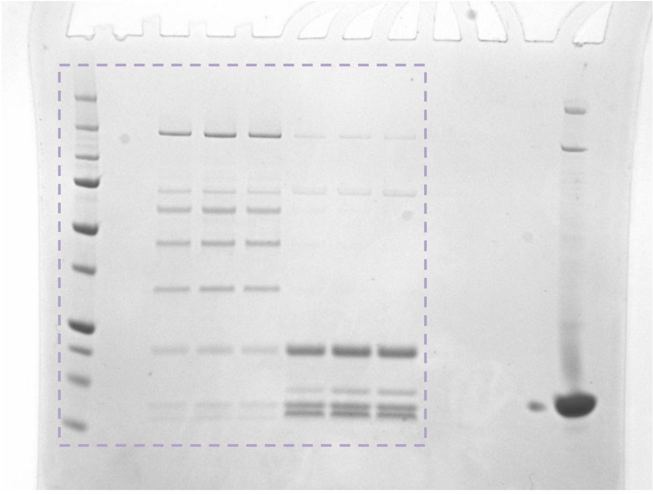

Fig. 3b

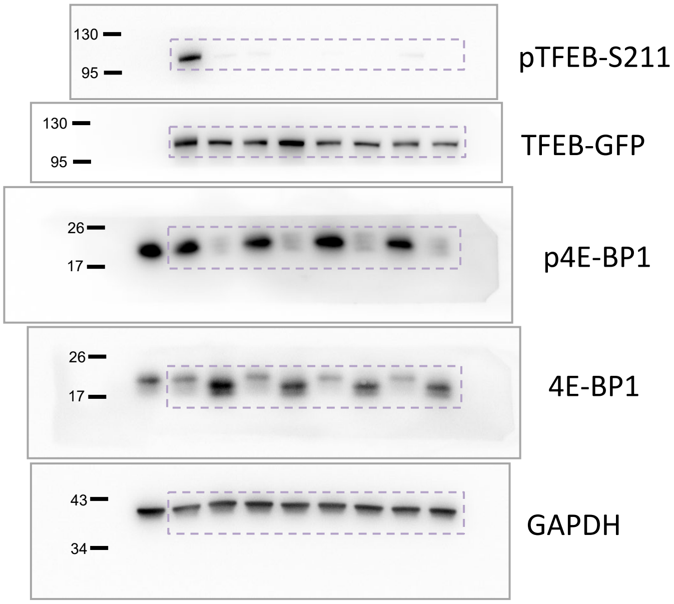

Fig. 3c

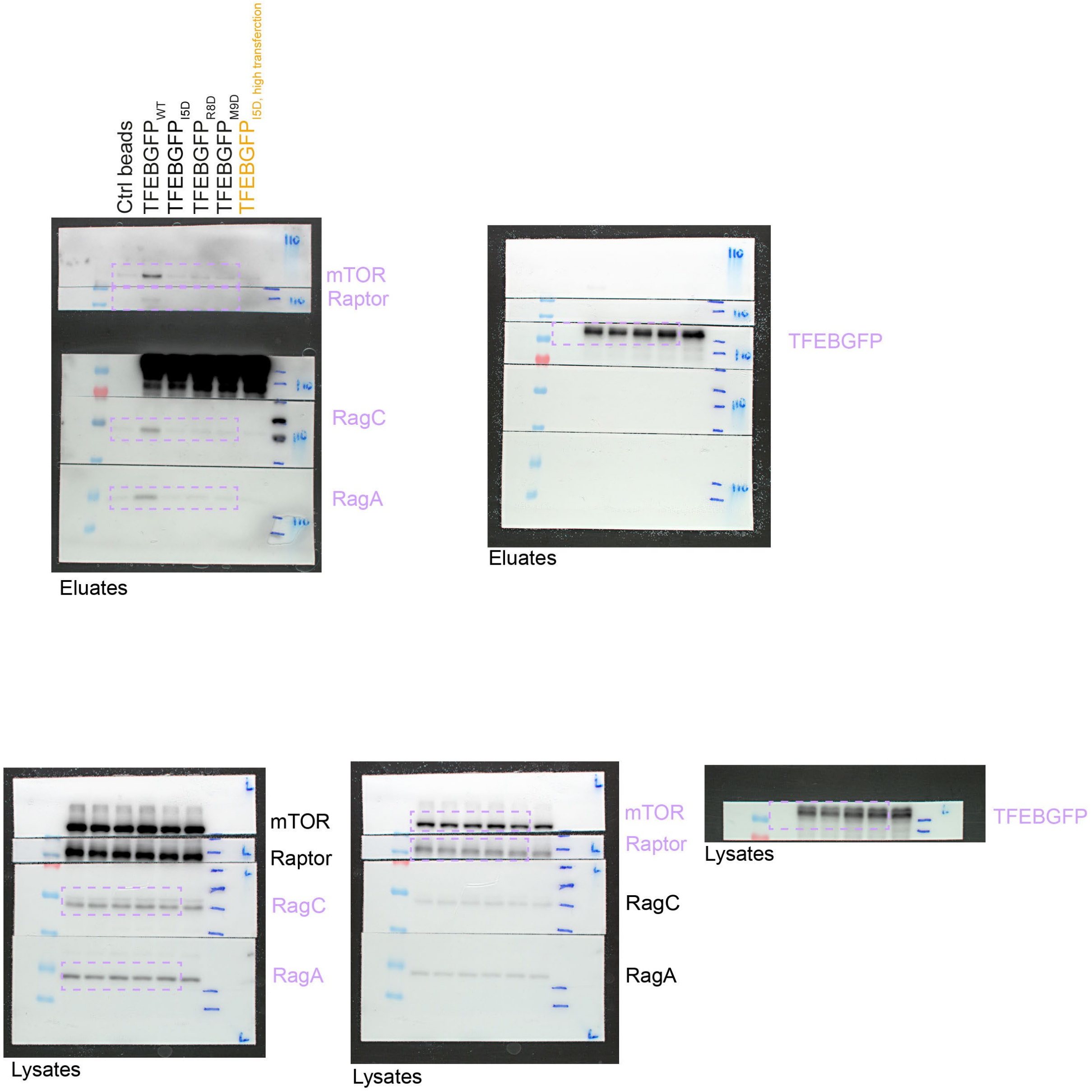
Ctrl beads
TFEBGFP WT
TFEBGFP I5D
TFEBGFP R8D
TFEBGFP M9D
TFEBGFP I5D, high transfection
mTOR
Raptor
RagC
RagA
Eluates
TFEBGFP
Eluates
mTOR
Raptor
RagC
RagA
Lysates
mTOR
Raptor
RagC
RagA
Lysates
TFEBGFP
Lysates

Not relevant

Fig. 3e

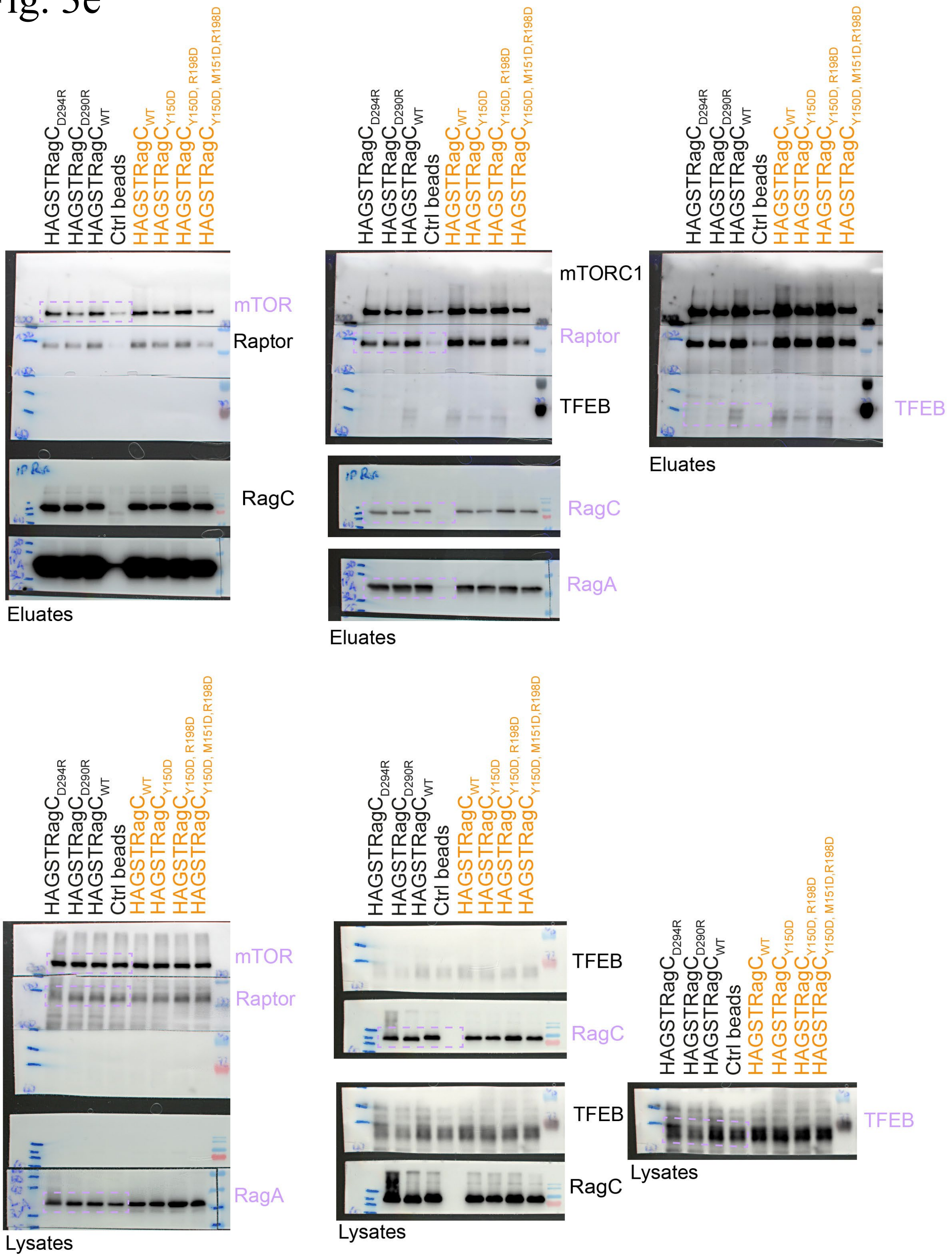

Blots are horizontally flipped in Fig. 3e

Fig. 3f

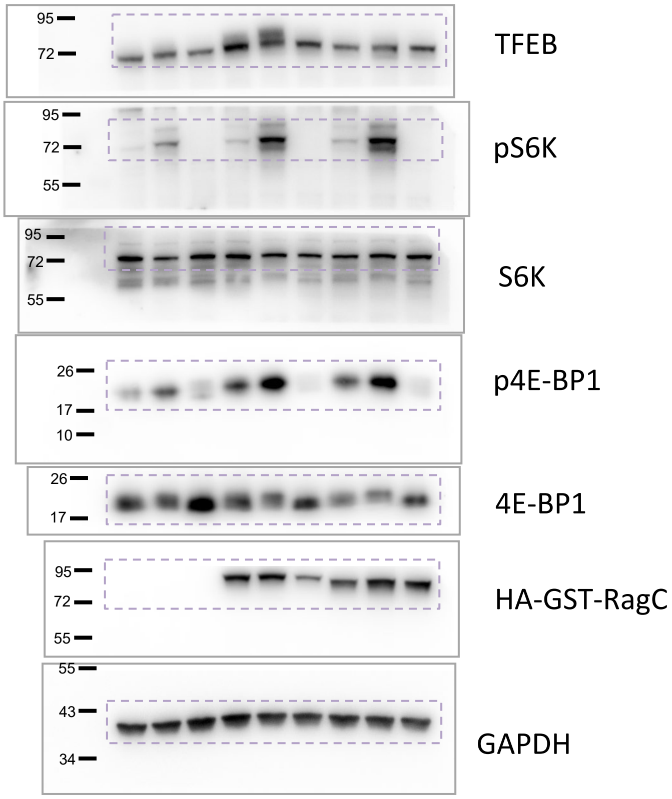

95
72
TFEB
95
72
55
pS6K
95
72
55
S6K
26
17
10
p4E-BP1
26
17
4E-BP1
95
72
55
HA-GST-RagC
55
43
34
GAPDH

Fig. 3g

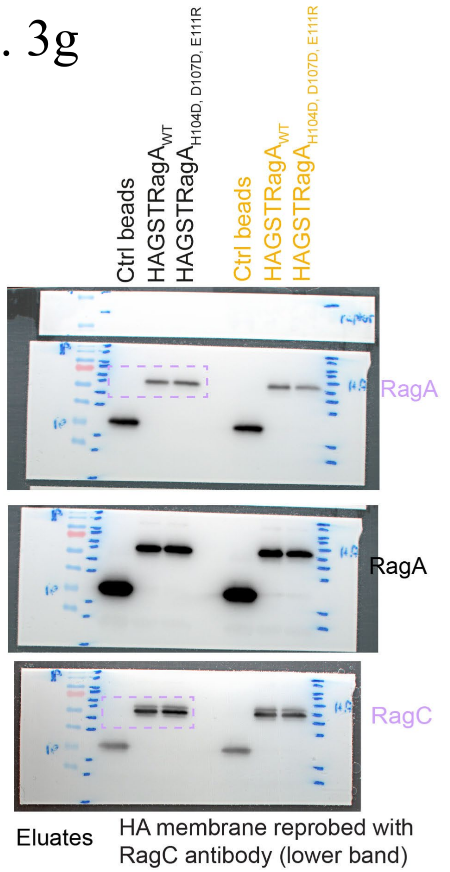

Ctrl beads
HAGSTRagA$_{WT}$
HAGSTRagA$_{H104D, D107D, E111R}$
Ctrl beads
HAGSTRagA$_{WT}$
HAGSTRagA$_{H104D, D107D, E111R}$
RagA
RagA
RagC
Eluates
HA membrane reprobed with
RagC antibody (lower band)

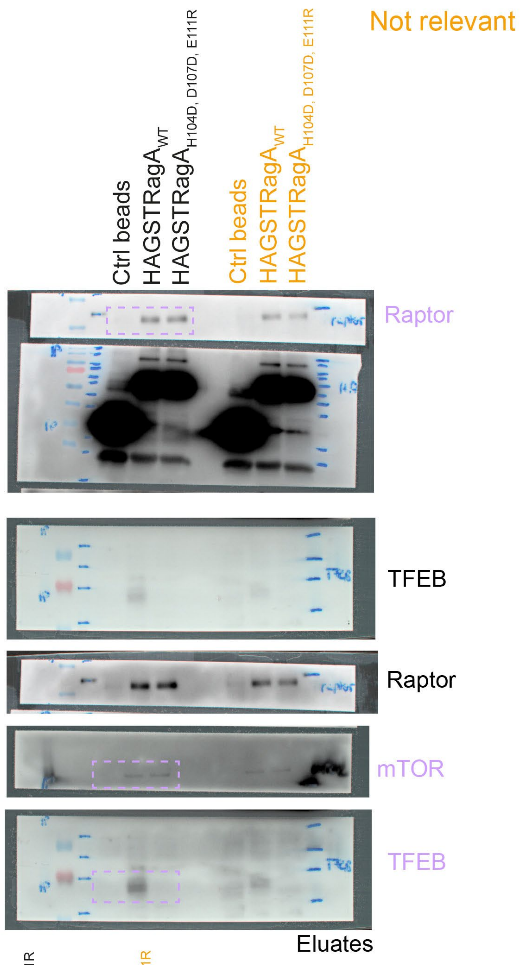

Not relevant
Ctrl beads
HAGSTRagA$_{WT}$
HAGSTRagA$_{H104D, D107D, E111R}$
Ctrl beads
HAGSTRagA$_{WT}$
HAGSTRagA$_{H104D, D107D, E111R}$
Raptor
TFEB
Raptor
mTOR
TFEB
Eluates

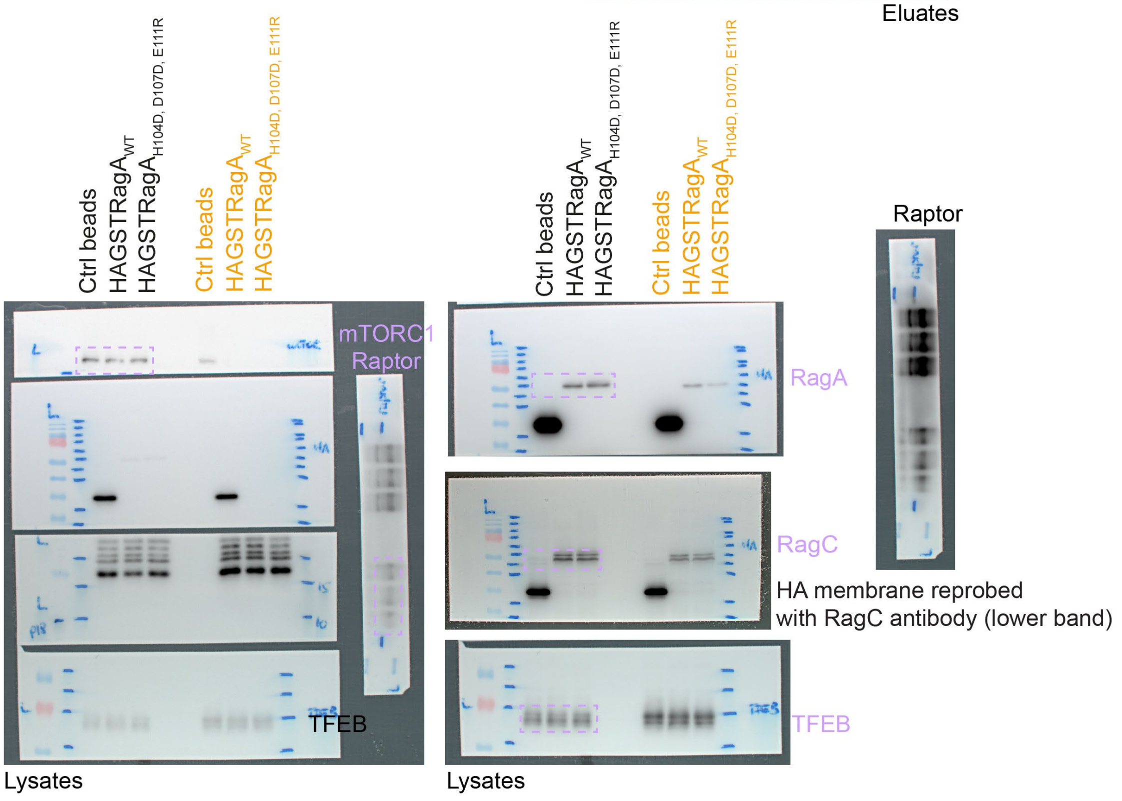

Ctrl beads
HAGSTRagA$_{WT}$
HAGSTRagA$_{H104D, D107D, E111R}$
Ctrl beads
HAGSTRagA$_{WT}$
HAGSTRagA$_{H104D, D107D, E111R}$
mTORC1
Raptor
TFEB
Lysates
Ctrl beads
HAGSTRagA$_{WT}$
HAGSTRagA$_{H104D, D107D, E111R}$
Ctrl beads
HAGSTRagA$_{WT}$
HAGSTRagA$_{H104D, D107D, E111R}$
Raptor
RagA
RagC
HA membrane reprobed
with RagC antibody (lower band)
TFEB
Lysates

Fig. 3h

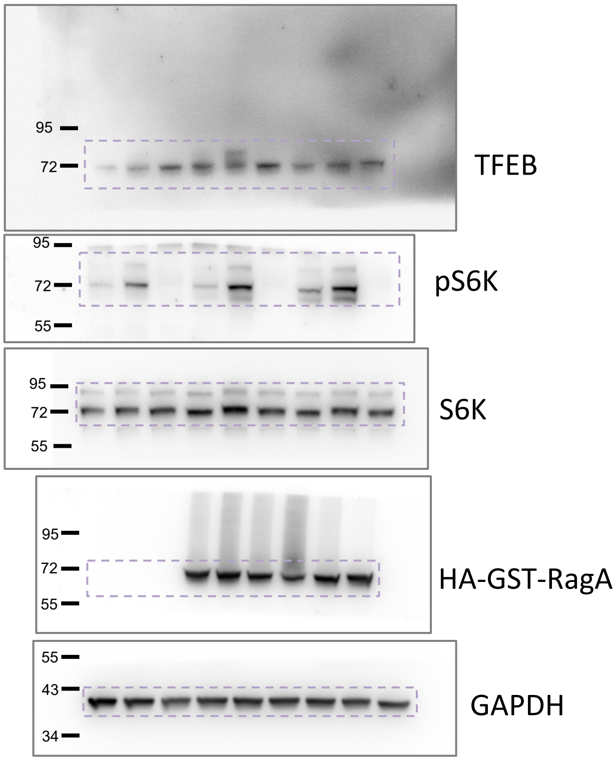
95
72
TFEB
95
72
55
pS6K
95
72
55
S6K
95
72
55
HA-GST-RagA
55
43
34
GAPDH

Fig. 4c

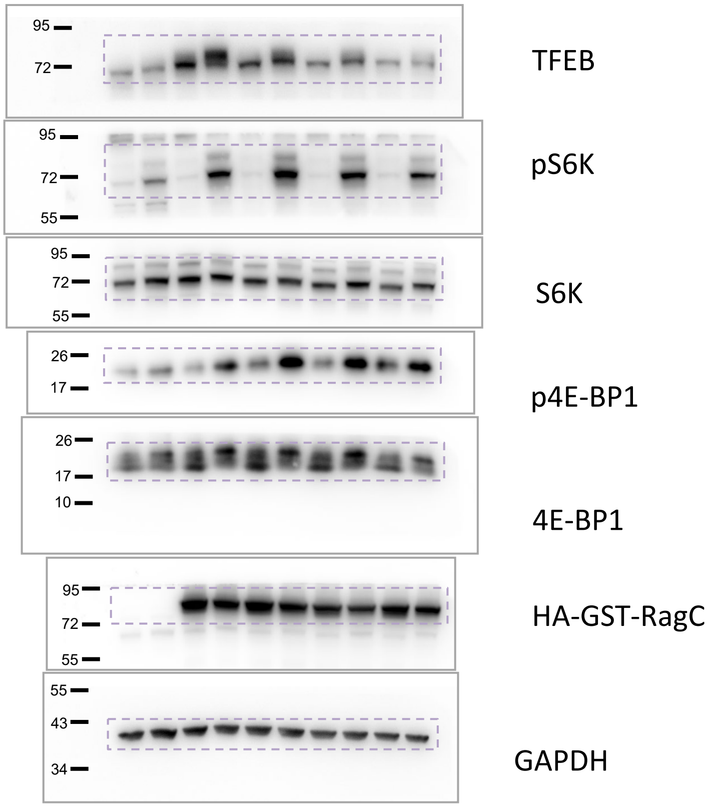
95
72
TFEB
95
72
55
pS6K
95
72
55
S6K
26
17
p4E-BP1
26
17
10
4E-BP1
95
72
55
HA-GST-RagC
55
43
34
GAPDH

Fig. 4d

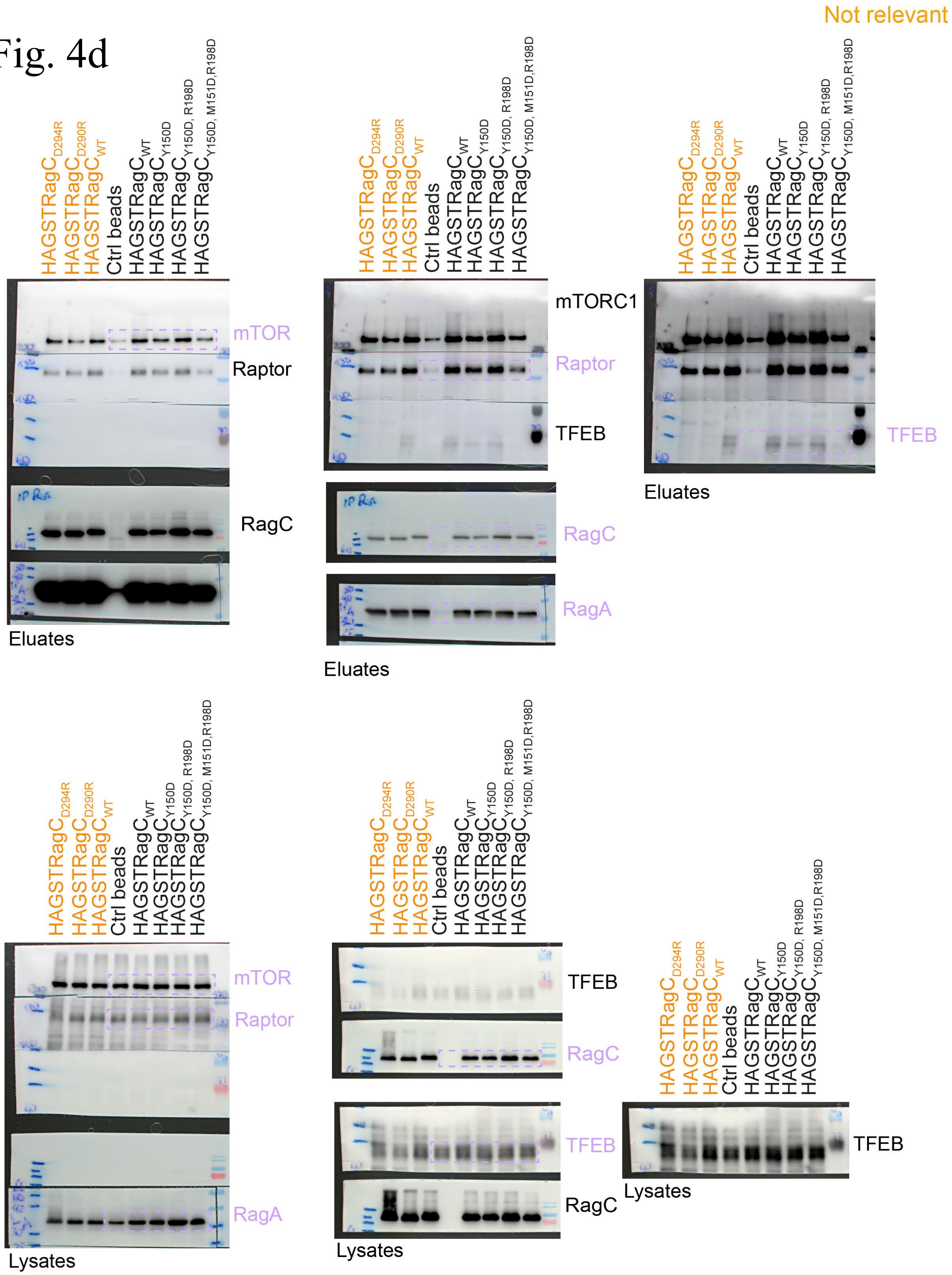

Not relevant
HAGSTRagC$_{D294R}$
HAGSTRagC$_{D290R}$
HAGSTRagC$_{WT}$
Ctrl beads
HAGSTRagC$_{WT}$
HAGSTRagC$_{Y150D}$
HAGSTRagC$_{Y150D, R198D}$
HAGSTRagC$_{Y150D, M151D,R198D}$
mTOR
Raptor
RagC
Eluates
HAGSTRagC$_{D294R}$
HAGSTRagC$_{D290R}$
HAGSTRagC$_{WT}$
Ctrl beads
HAGSTRagC$_{WT}$
HAGSTRagC$_{Y150D}$
HAGSTRagC$_{Y150D, R198D}$
HAGSTRagC$_{Y150D, M151D,R198D}$
mTORC1
Raptor
TFEB
RagC
RagA
Eluates
HAGSTRagC$_{D294R}$
HAGSTRagC$_{D290R}$
HAGSTRagC$_{WT}$
Ctrl beads
HAGSTRagC$_{WT}$
HAGSTRagC$_{Y150D}$
HAGSTRagC$_{Y150D, R198D}$
HAGSTRagC$_{Y150D, M151D,R198D}$
TFEB
Eluates
HAGSTRagC$_{D294R}$
HAGSTRagC$_{D290R}$
HAGSTRagC$_{WT}$
Ctrl beads
HAGSTRagC$_{WT}$
HAGSTRagC$_{Y150D}$
HAGSTRagC$_{Y150D, R198D}$
HAGSTRagC$_{Y150D, M151D,R198D}$
mTOR
Raptor
RagA
Lysates
HAGSTRagC$_{D294R}$
HAGSTRagC$_{D290R}$
HAGSTRagC$_{WT}$
Ctrl beads
HAGSTRagC$_{WT}$
HAGSTRagC$_{Y150D}$
HAGSTRagC$_{Y150D, R198D}$
HAGSTRagC$_{Y150D, M151D,R198D}$
TFEB
RagC
TFEB
RagC
Lysates
HAGSTRagC$_{D294R}$
HAGSTRagC$_{D290R}$
HAGSTRagC$_{WT}$
Ctrl beads
HAGSTRagC$_{WT}$
HAGSTRagC$_{Y150D}$
HAGSTRagC$_{Y150D, R198D}$
HAGSTRagC$_{Y150D, M151D,R198D}$
TFEB
Lysates

Extended Data Fig. 1b

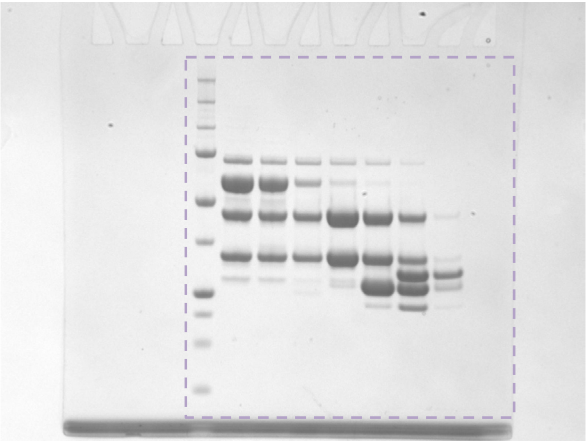

Extended Data Fig. 1c

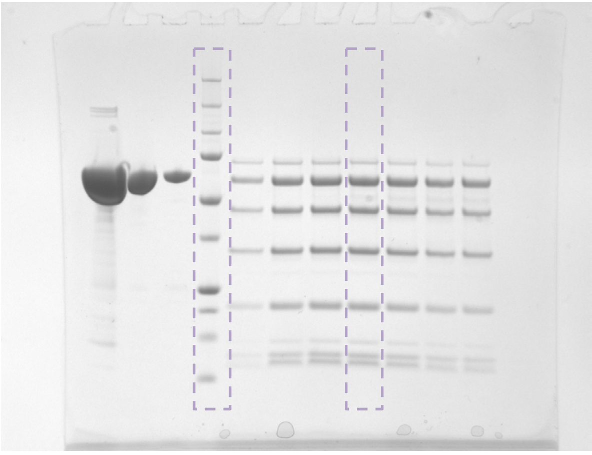

Extended Data Fig. 5a

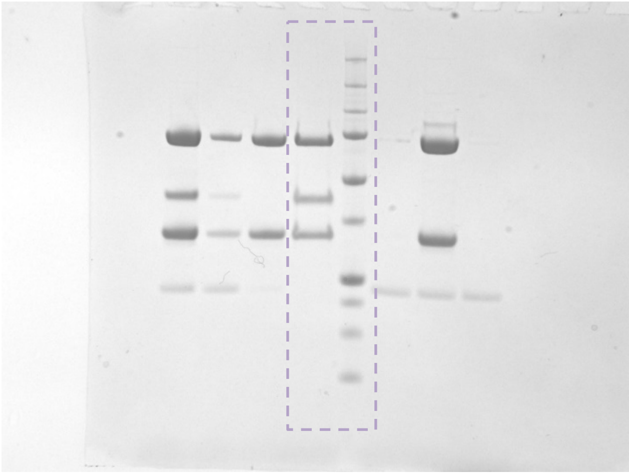

Extended Data Fig 5b

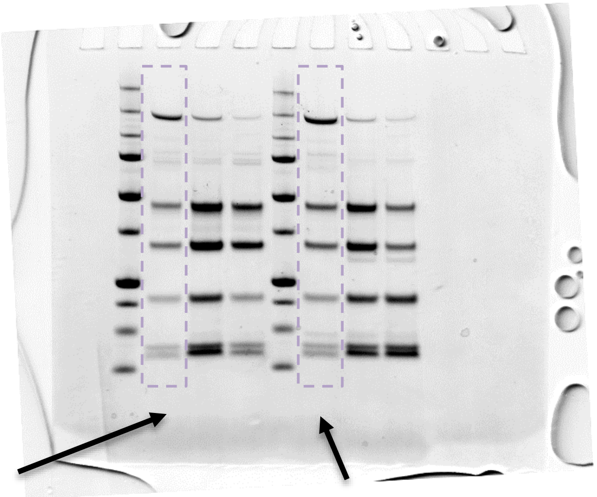

Raptor-Rag-Ragulator Raptor-TFEB$^{1-109}$-Rag-Ragulator

Extended Data Fig. 7e

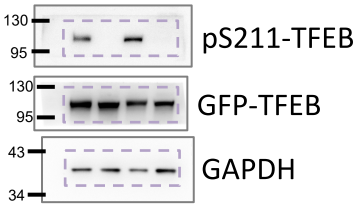

Extended Data Fig. 8c

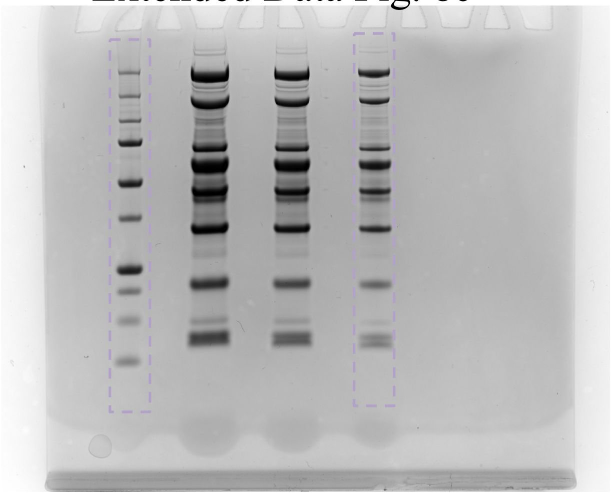

Supplementary Fig. 2: Wild-type TFEB does not copurify with Rags.
SDS-PAGE analysis of the purification for TFEB (wild-type) with co-expressed active Rags is shown on the left. The right panel for purification of TFEB (S211A/NLS) with co-expressed active Rags is shown for comparison. The purification is done with the GST tag on RagC before TEV cleavage. TFEB (wild-type)-GFP was not co-purified with active Rags.

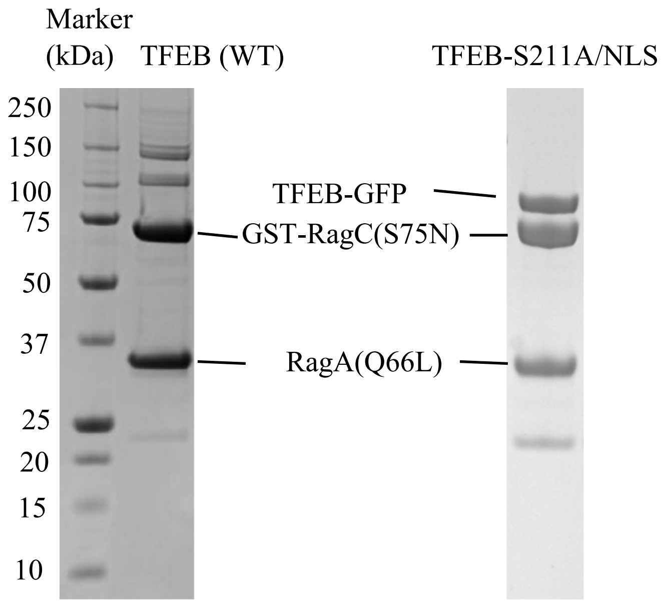

Supplement: Supplementary file 1 — This file contains Supplementary Fig. 1: Original images of SDS–PAGE and immunoblots and Supplementary Fig. 2: Wild-type TFEB does not copurify with Rags. [file 41586_2022_5652_MOESM1_ESM.pdf]
