# Supplementary material for: Structure of the lysosomal mTORC1–TFEB–Rag–Ragulator megacomplex
Source: Nature. 2023 Jan 25;614(7948):572–9. doi: 10.1038/s41586-022-05652-7 (PMC9931586; doi:10.1038/s41586-022-05652-7)
Supplement: Supplementary file 2 — Reporting Summary [file 41586_2022_5652_MOESM2_ESM.pdf]

nature portfolio

Corresponding author(s): James H. Hurley

Last updated by author(s): May 2, 2022

Reporting Summary

Nature Portfolio wishes to improve the reproducibility of the work that we publish. This form provides structure for consistency and transparency in reporting. For further information on Nature Portfolio policies, see our Editorial Policies and the Editorial Policy Checklist.

Statistics

For all statistical analyses, confirm that the following items are present in the figure legend, table legend, main text, or Methods section.

n/a	Confirmed	
☒	☐	The exact sample size (n) for each experimental group/condition, given as a discrete number and unit of measurement
☒	☐	A statement on whether measurements were taken from distinct samples or whether the same sample was measured repeatedly
☒	☐	The statistical test(s) used AND whether they are one- or two-sided<br>*Only common tests should be described solely by name; describe more complex techniques in the Methods section.*
☒	☐	A description of all covariates tested
☒	☐	A description of any assumptions or corrections, such as tests of normality and adjustment for multiple comparisons
☒	☐	A full description of the statistical parameters including central tendency (e.g. means) or other basic estimates (e.g. regression coefficient) AND variation (e.g. standard deviation) or associated estimates of uncertainty (e.g. confidence intervals)
☒	☐	For null hypothesis testing, the test statistic (e.g. F, t, r) with confidence intervals, effect sizes, degrees of freedom and P value noted<br>*Give P values as exact values whenever suitable.*
☒	☐	For Bayesian analysis, information on the choice of priors and Markov chain Monte Carlo settings
☒	☐	For hierarchical and complex designs, identification of the appropriate level for tests and full reporting of outcomes
☒	☐	Estimates of effect sizes (e.g. Cohen's d, Pearson's r), indicating how they were calculated

Our web collection on statistics for biologists contains articles on many of the points above.

Software and code

Policy information about availability of computer code

Data collection	SerialEM, MotionCor2, CryoSPARC v3, Relion3
Data analysis	Relion 3, Chimera X, COOT, ISOLDE

For manuscripts utilizing custom algorithms or software that are central to the research but not yet described in published literature, software must be made available to editors and reviewers. We strongly encourage code deposition in a community repository (e.g. GitHub). See the Nature Portfolio guidelines for submitting code & software for further information.

Data

Policy information about availability of data

All manuscripts must include a data availability statement. This statement should provide the following information, where applicable:

- Accession codes, unique identifiers, or web links for publicly available datasets
- A description of any restrictions on data availability
- For clinical datasets or third party data, please ensure that the statement adheres to our policy

Structural coordinates have been deposited in the RCSB and EM density in EMDB and the accession codes have been provided. We have requested their release as soon as possible.

Field-specific reporting

Please select the one below that is the best fit for your research. If you are not sure, read the appropriate sections before making your selection.

☒ Life sciences ☐ Behavioural & social sciences ☐ Ecological, evolutionary & environmental sciences

For a reference copy of the document with all sections, see nature.com/documents/nr-reporting-summary-flat.pdf

Life sciences study design

All studies must disclose on these points even when the disclosure is negative.

Sample size	N/A
Data exclusions	N/A
Replication	at least three replicates for cellular assays
Randomization	N/A
Blinding	N/A

Reporting for specific materials, systems and methods

We require information from authors about some types of materials, experimental systems and methods used in many studies. Here, indicate whether each material, system or method listed is relevant to your study. If you are not sure if a list item applies to your research, read the appropriate section before selecting a response.

Materials & experimental systems

n/a	Involved in the study	
☐	☒	Antibodies
☐	☒	Eukaryotic cell lines
☒	☐	Palaeontology and archaeology
☒	☐	Animals and other organisms
☒	☐	Human research participants
☒	☐	Clinical data
☒	☐	Dual use research of concern

Methods

n/a	Involved in the study	
☒	☐	ChIP-seq
☒	☐	Flow cytometry
☒	☐	MRI-based neuroimaging

Antibodies

Antibodies used	Antibodies to mTOR (Cat# 2983 - 1:100 IF) Phospho-p70 S6 Kinase (Thr389) (1A5) (Cat# 9206 - 1:1000 WB), p70 S6 Kinase (Cat# 9202 - 1:1000 WB), 4E-BP1 (Cat# 9644 - 1:1000 WB), Phospho-4E-BP1 (Ser65) (Cat# 9456 - 1:1000 WB), TFEB (Cat# 4240 - 1:1000 WB), Phospho-TFEB S211 (Cat# 37681 – 1:1000 WB) were from Cell Signaling Technology; antibodies to GAPDH (6C5) (Cat# sc-32233 - 1:15000 WB) and LAMP-1 (H4A3) (Cat# sc-20011 - 1:500 IF) were from Santa Cruz; antibody to HA.11 Epitope Tag (Cat# 901513) was from Biolegend; HRP-conjugated secondary antibodies to Mouse (Cat# 401215 - 1:5000 dilution) and Rabbit (Cat# 401315 - 1:5000 dilution) IgGs were form Calbiochem.
Validation	Manufacturer's websites, see above

Eukaryotic cell lines

Policy information about cell lines

Cell line source(s)	HeLa
Authentication	Cell lines were validated by morphological analysis and routinely tested for absence of mycoplasma
Mycoplasma contamination	Cell lines were routinely tested for absence of mycoplasma.
Commonly misidentified lines (See ICLAC register)	N/A

March 2021
